# Spatial optimization of residential care facility locations in Beijing, China: maximum equity in accessibility

**DOI:** 10.1186/1476-072X-13-33

**Published:** 2014-09-01

**Authors:** Zhuolin Tao, Yang Cheng, Teqi Dai, Mark W Rosenberg

**Affiliations:** 1 School of Geography, Beijing Normal University, 19 Xinjiekouwai Avenue, Beijing 100875, China; 2 Department of Geography, Queen’s University, Kingston, Ontario K7L 3N6, Canada

**Keywords:** Spatial optimization, Residential care facilities, Equity, Accessibility, Quadratic programming, PSO algorithm

## Abstract

**Background:**

The residential care system is rapidly developing and plays an increasingly important role in care for the elderly in Beijing. A noticeable disparity in the accessibility to existing residential care facilities, however, is demonstrated in existing studies. The spatial optimization of residential care facility (RCF) locations is urgently needed to promote equal access to residential care resources among the elderly population.

**Methods:**

A two-step floating catchment area method with an additional distance-decay function is adopted to measure accessibility to residential care facilities. The spatial optimization model is developed to maximize equity in accessibility by minimizing the total square difference between the accessibility score of each demand location and the weighted average accessibility score. The Particle Swarm Optimization (PSO) method is implemented for the solution.

**Results:**

The optimized RCF layouts improve equal spatial access to residential care resources with very low accessibility standard variation (0.0066). A relatively large number of beds (51% of the total beds) to be located in the suburban districts between the central and periphery districts of Beijing are optimized. A smaller number of beds to be located in the central and periphery districts (33% and 16% respectively) are optimized. The gaps between the existing and optimized layouts suggest that more RCF beds (5961 beds) are needed in suburban districts, while the RCF beds in some subdistricts located in the central and periphery districts are oversupplied (5253 and 1584 surplus beds respectively).

**Conclusions:**

The optimized results correspond to the municipal special plan proposed by the Beijing government. The optimization objective of this study is different from traditional facility location optimization models, and the method is efficient in maximizing equal access to residential care facilities. This method can support knowledge-based policy-making and planning of residential care facilities.

## Background

The elderly population in China reached 178 million in 2010, accounting for 13.3 percent of its total population. The Chinese government implemented the one-child policy in the 1980s, which has accelerated the population aging process. Beijing, the capital of China, also faces the challenge of population aging, even with the massive influx of younger working age people taking place. The elderly population (aged 60 and over) was 1.7 million in 2000 in Beijing (12.5% of the total population), and it rapidly increased to 2.63 million (20.3% of the total population) in 2012 [1]. The number of the elderly population is expected to be 4.15 million (30% of the total population) in 2025 [2]. Care for the elderly population has becomes a critically important issue for the central government of China, the local government of Beijing and Chinese society.

The socio-cultural changes in contemporary China have meant new challenges for the traditional family care system for elderly people. A residential care system is rapidly developing and will play an increasingly important role in care for the elderly population in the future [3-7]. *The Construction Plan of Social Elderly Care System (2011–2015)*[8] issued by the State Council of the People’s Republic of China advocated the development of an elderly care system that mainly depends on traditional family and community care, supplemented by residential care. The Beijing local government has issued its first municipal special plan for the residential care system, which aims to provide residential care services for four percent of the elderly population in Beijing by 2020 [9]. There is still a large gap between this policy goal and the existing residential care resources in Beijing. The number of beds offered by RCFs was 69.3 thousand in 2013 [10]. To reach the policy goal, another 10,000 beds need to be added per year to the existing stock of beds from 2014 to 2020.

To distribute public services equitably and efficiently are the primary goals of the government. Accessibility to RCFs, however, shows a noticeable disparity among the various regions in Beijing [11,12], which reflects the irrational locations of RCFs. Therefore, the spatial optimization of RCFs locations is urgently needed to maximize equal access to RCFs for the elderly population living across Beijing.

Existing research on residential care has mainly focused on the development of residential care services [13-15], spatial distribution of RCFs [16,17], characteristics of older people living in RCFs [4,18], preference for RCFs [19], and access to RCFs [11,12,20]. Little attention, however, has been paid to spatial optimization of RCFs locations in the context of Beijing. Some studies have examined the optimization of other types of facilities (e.g., health care facility) locations [21-25]. The optimization objectives of the classic location-allocation models, however, mainly focus on efficiency rather than equity of facility locations [25,26]. Wang and Tang propose a model to deal with equity of accessibility to facilities, which aims to minimize the disparity in accessibility [26].

This paper aims to develop a spatial optimization model for the locations of RCFs to maximize equal access in the context of Beijing. The optimized locations of RCFs demonstrates one way forward for knowledge-based planning of residential care resources in Beijing. Section Two of this paper introduces the measurement of accessibility to RCFs. Following that, a spatial optimization model is developed to consider accessibility. In Section Three, the Particle Swarm Optimization Approach (PSO) is introduced as a solution to the optimization problem. The results of the PSO approach are presented in Section Four and the implications for planning and policy are discussed in Section Five.

### The measurement of accessibility to RCFs and the spatial optimization model

#### ***Measurement of accessibility to RCFs***

Access to facilities has various definitions. One of the popular definitions classifies access as either spatial access or aspatial access. Spatial access emphasizes the spatial separation between supply and demand as a barrier or a facilitator, while aspatial access stresses nongeographic barriers or facilitators [27]. This study focuses on spatial accessibility of RCFs.

There are various methods to measure spatial accessibility to facilities, among which the two-step floating catchment area (2SFCA) method and the gravity-based method are frequently used. Wang summarizes various indices of accessibility in a general formulation [25]:

(1) $$A_{i}=\sum_{j=1}^{n}S_{j}f\left(d_{\mathrm{ij}}\right)/\sum_{k=1}^{m}D_{k}f\left(d_{\mathrm{kj}}\right)$$

where *A*_*i*_ is the accessibility at demand point *i*, *S*_*j*_ is the capacity of supply at location *j*, *D*_*k*_ is the demand (i.e. elderly population in this paper), *d*_*ij*_ (*d*_*kj*_) is the distance or travel time between *i*(*k*) and *j*, *f* is a general distance-decay function. Specifically, *A*_*i*_ refers to the number of accessible RCF beds per elderly person of the demand point *i*.

With regard to the distance-decay function *f*, various forms (either discrete or continuous) can be taken. The 2SFCA, for example, takes a dichotomous form, that is, any travel time within a threshold is equally accessible and any travel time beyond the threshold is inaccessible. By contrast, the gravity-based index takes a power function form. There are several other forms in the existing literature [25].

The study conducted by Cheng, Wang and Rosenberg uses 2SFCA to measure accessibility to RCFs in Beijing [11]. As mentioned earlier, 2SFCA treats travel time within the catchment area equally. Our previous research and the study conducted by Gao et al., however, found that the distance between RCFs and families’ locations is one of the most important factors in the decision-making process for choosing a suitable RCF by elderly people and their family members [19,20]. The original 2SFCA method ignores accessibility differences within the catchment area. Adding a distance-decay function within the catchment area can improve the performance of 2SFCA, thus the function *f* in formula (1) follows the formulation:

(2) $$f\left(d_{\mathrm{ij}}\right)=\left\{\begin{array}{c} d_{\mathrm{ij}}^{-\beta }\;,\;d_{\mathrm{ij}}\leq d_{0}\\ \;0,\;d_{\mathrm{ij}}>d_{0} \end{array}\right)$$

where *d*_*ij*_ is the distance or travel time between *i* and *j*, *d*_0_ is the threshold distance (i.e. the catchment size), and *β* is the distance-decay parameter.

As summarized by Peeters and Thomas, the distance-decay parameter *β* in existing studies lies between 0.9 and 2.29 [28]. Wang and Zhang conducted a sensitive analysis on *β*, setting *β* as 1 or 2 respectively [29]. The results show that 2 is better for measuring the accessibility of health care facilities [29,30]. However, it is different for RCFs compared to other health care facilities. The service users of RCFs travel much less frequently than the service users of health care facilities. Therefore, the distance-decay for RCFs is weaker than for other health care facilities, especially for emergency medical services. That is to say, *β* value for RCFs should be smaller than for other health care facilities. Our previous study measuring spatial accessibility to RCFs in Beijing sets *β* as 1 [12]. In this study, sensitivity analysis of the value of *β* will first be conducted, with various *β* values from 0.6 to 1.4.

The catchment size *d*_0_ differs among various facilities and regions. Cheng found the maximum travel time that the elderly population and their families prefer is 1 to 1.5 hours in Beijing [3]. This study sets *d*_0_ as 1 hour.

#### ***Formulation of the spatial optimization model***

The classic location-allocation models focus on the efficient layout of facilities, such as maximum coverage (the maximum covering location problem), minimum number of facilities (the location set covering problem), and minimum travel time from demand points to facilities (the p-median problem) [21-25].

Little research has taken equity as the goal in optimizations of facility locations. One of the important reasons is that equal accessibility is often impractical and sometimes even unreachable in planning [25,26]. Wang and Tang propose a model to deal with equity of accessibility to facilities, which aims to minimize the inequality of accessibility [26]. The model is written as:

(3) $$\text{minimize}\sum_{i=1}^{m}{\left(A_{i}-a\right)}^{2}\text{,}$$

(4) $$a=\sum_{i=1}^{m}\frac{D_{i}}{D}A_{i}=\frac{S}{D}\;\text{,}$$

where *A*_*i*_ is the accessibility score calculated by formulas (1) and (2), *a* is the weighted average accessibility, *S* is the total supply and *D* is the total demand. This model is a non-linear programming problem, which is different from the traditional linear programming models.

#### ***Study area and data processing***

**Figure 1:**
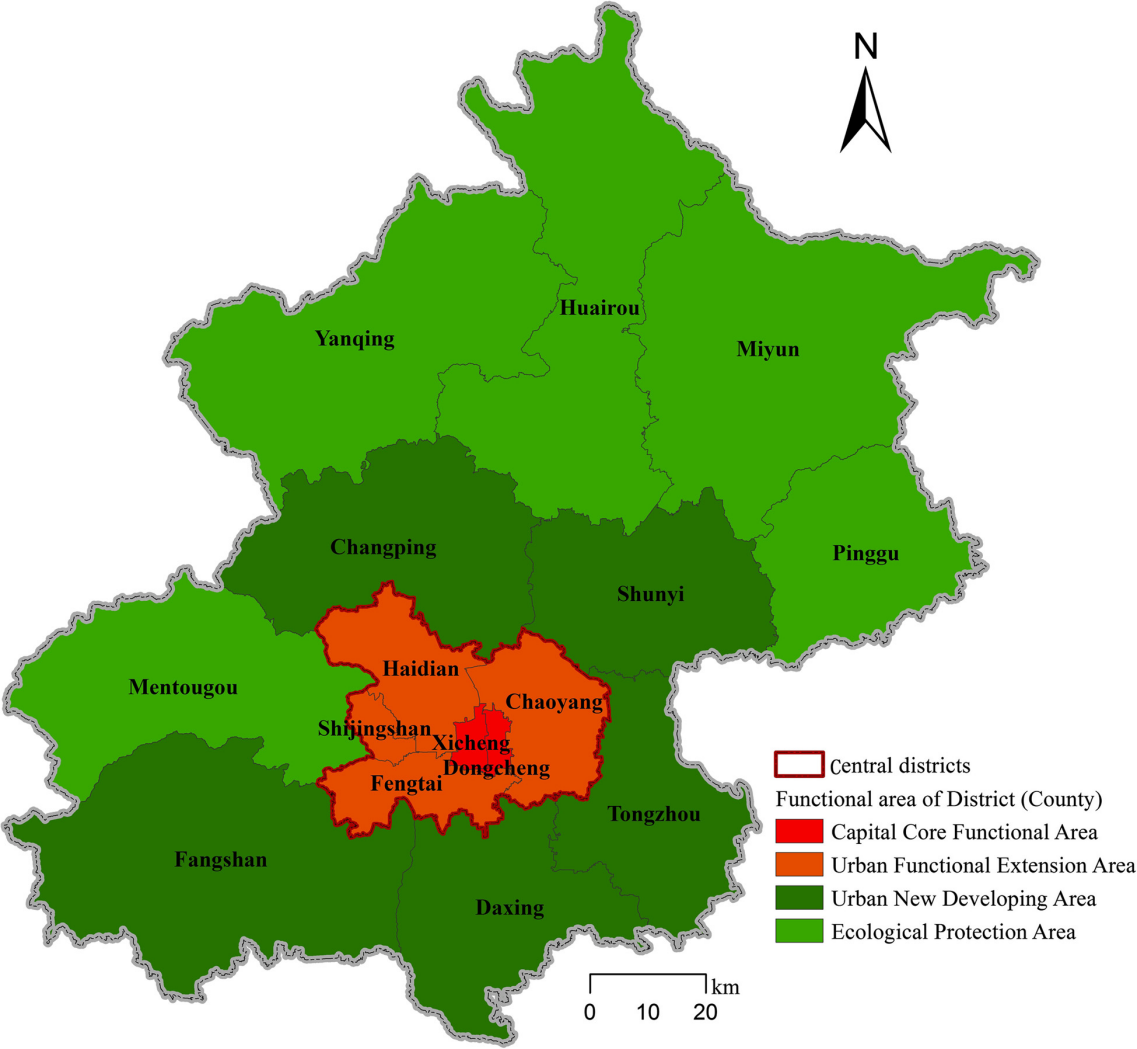
The functional area of 14 districts and 2 counties of Beijing.

There are 14 districts and 2 counties in Beijing (Figure 1). They are divided into four functional areas according to the *Beijing Urban Master Plan (2004–2020)*. The Capital Core Functional Area includes Dongcheng and Xicheng Districts, and the Urban Functional Extension Area includes Chaoyang, Haidian, Fengtai and Shijingshan Districts. The central districts of Beijing include the six districts in these two functional areas. The suburban districts are mainly included in the Urban New Developing Area (5 districts), and the peripheral districts are mainly included in the Ecological Protection Area (3 districts and 2 counties).

**Figure 2:**
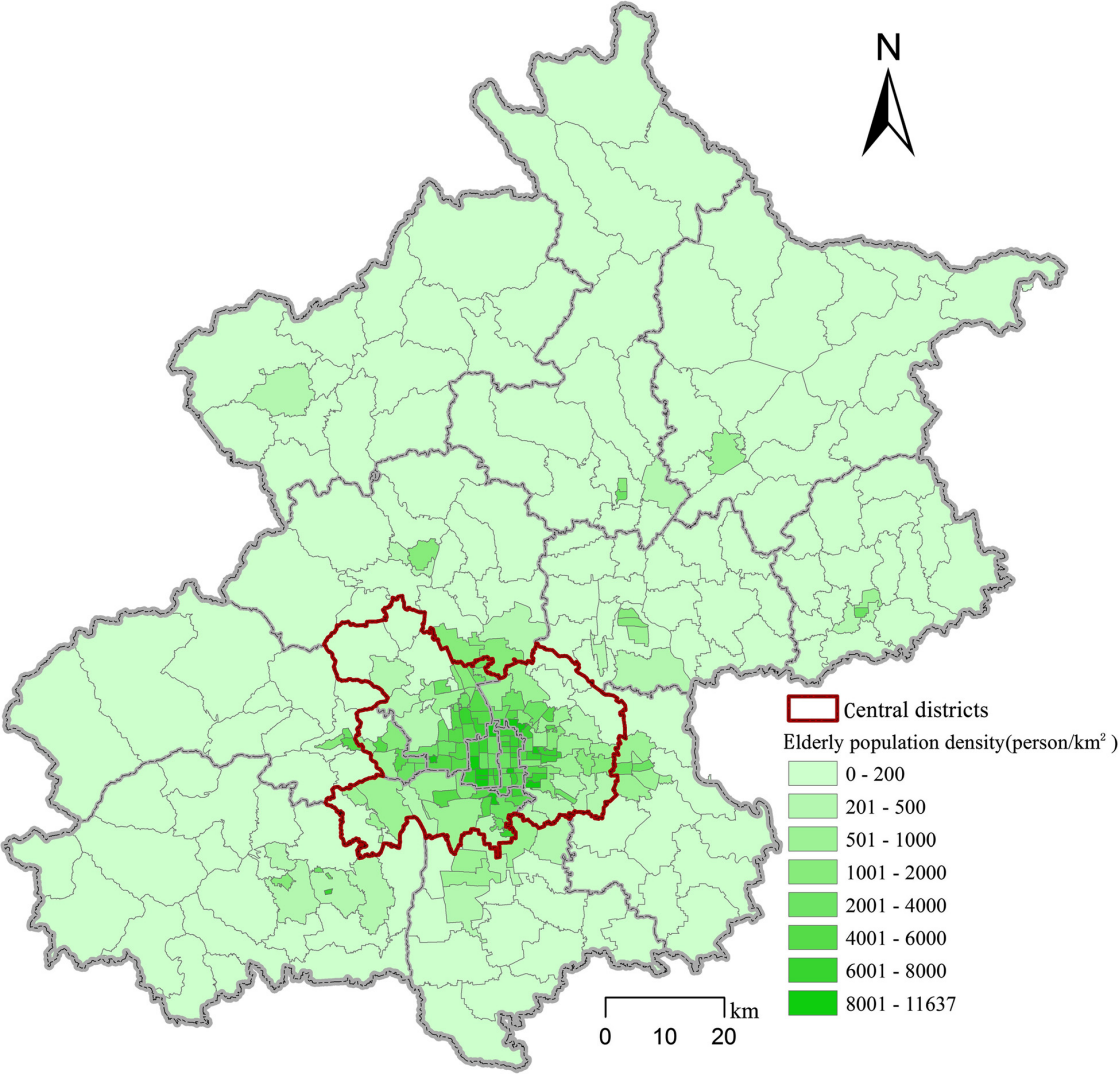
The spatial distribution of elderly population at the subdistrict level in Beijing in 2010.

The elderly population data at the subdistrict level is from the 6^th^ population census. In the census, there are 324 subdistrict administrative units (Streets within urban areas and villages or townships in suburban and peripheral districts). We take the 324 units as the demand locations and their elderly population as the measure of demand. The spatial distribution of the elderly population shows an uneven pattern, with a decreased elderly population outwards as the distance increases from the city center (Figure 2). Locations of the subdistrict level units are obtained from the centroids of their administrative boundaries by using ArcGIS software.

The candidate facility locations are determined at the subdistrict level as suggested by the municipal special plan for the residential care system. We measure the travel time from the demand locations to the candidate facility locations based on the road network of Beijing in 2010. To improve the accuracy of travel time, we adopt the estimated driving speeds of the road network in Beijing [31]. The speeds of four-level roads are set at 50, 40, 30 and 20 km/h respectively. The network analysis tool in ArcGIS is used to calculate the shortest travel time from each demand location to each candidate facility location.

### The Particle Swarm Optimization Approach (PSO)

The optimization model is a quadratic programming (QP) problem as shown in formulas 3 and 4. A complex transformation is necessary to prepare for the implementation of QP. QP is usually inefficient and time-consuming and it might not be feasible for large computational problems. Thus, the Particle Swarm Optimization Approach (PSO) is adopted as a solution to the limitations of QP.

#### ***Introduction to the PSO***

The PSO is an optimization algorithm originally proposed by Kennedy and Eberhart [32], analogous to the foraging behavior of birds flocking together. The algorithm has been widely used in various fields [33]. Notably, the PSO can be applied for solving location-allocation problems [34].

The PSO characterizes a population as m particles and potential solutions. Each particle is treated as a point in an n-dimensional bounded solution and is initially assigned a randomized velocity. Thus any particle *i* has a position $X_{i}^{t}$ and a velocity $V_{i}^{t}$ at time *t*. Two best positions are introduced: the local best position *Pbest*_*i*_ as the best of all the positions of particle *i* (i.e. $\text{Pbes}t_{i}\in \left\{X_{i}^{1},\;X_{i}^{2}\;,\;\ldots ,\;X_{i}^{t}\right\}$.) and the global best position *Gbest* as the best position among all particles (i.e. *Gbest* ∈ {*Pbest*_1_, *Pbest*_2_, …, *Pbest*_*m*_} from the beginning time. Each time each particle updates its position according to its previous position and the current velocity. The velocity is updated based on the previous velocity and the gap between the previous position, the local best position and the global best position. The process is described as follows:

(5) $$V_{i}^{t+1}=\omega V_{i}^{t}+c_{1}r_{1}\left(\text{Pbes}t_{i}^{t}-X_{i}^{t}\right)+c_{2}r_{2}\left(\text{Gbes}t^{t}-X_{i}^{t}\right)$$

(6) $$X_{i}^{t+1}=X_{i}^{t}+V_{i}^{t+1}$$

where *ω* is the inertia weight, *c*_1_ and *c*_2_ are acceleration constants, and *r*_1_ and *r*_2_ are random numbers between 0 and 1.

In addition, a fitness function is introduced to evaluate the performance of each particle. The fitness function can be defined according to the user’s needs. To solve the optimization model in Section Two, the fitness function in this paper is defined as the total squared difference between the accessibility score of each demand location and weighted average accessibility:

(7) $$\text{fitness}=\sum_{i=1}^{m}{\left(A_{i}-a\right)}^{2}\text{,}$$

where *A*_*i*_ and *a* are given by formulas (1) and (4), respectively.

Besides the fitness function adopted in this study, the PSO solution allows various fitness function forms, such as ordinary least square (OLS), minimizing the maximum absolute error, and minimizing the sum of the absolute deviations [34], which is an important advantage compared to the traditional nonlinear programming solution.

#### ***Implementation***

We implement the PSO solution by using the PSO toolbox in MATLAB. The parameters are set based on the proposed values in the toolbox. The number of particles is 36 and the maximum number of iterations is 3000 (Table 1), aiming to obtain a more feasible solution. In addition, to assure a total supply *S* of all the candidate facility locations, we set the dimension of each particle as 323 ({*P*_1_, *P*_2_, …, *P*_323_}) then the remaining dimension *P*_324_ = *S* - *P*_1_ - *P*_2_-, …, - *P*_323_

**Table 1:** The parameters in the PSO solution

**Parameter**	**Value**
Number of particles	36
Maximum number of iterations	3000
Acceleration constants c_2_	2
Range of X	[0,2500]
Maximum velocity	0.2*Range
Dimension of particles	323

## Results

### Optimization results

**Figure 3:**
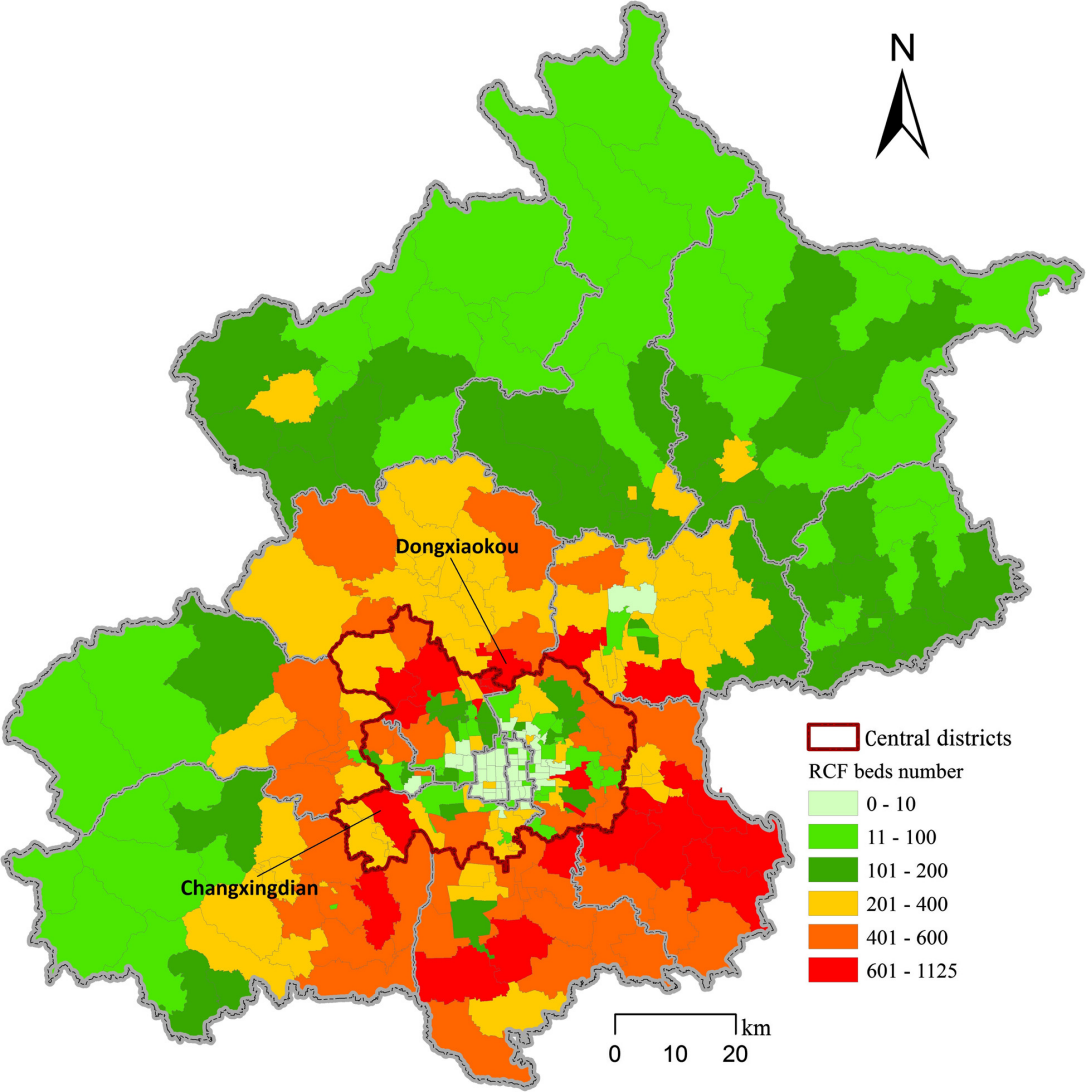
The spatial distribution of the optimized residential care resources at subdistrict level in Beijing.

We calculated five optimization scenarios where *β* ranges from 0.6 to 1.4 to assess the sensitivity of our results. With each *β* value, the accessibility to RCFs is measured based on the actual distribution of RCFs in Beijing. Both the standard variations of actual and equalized accessibility to RCFs are calculated, and the ratio between them reflects the efficiency of optimization (Table 2). The larger a *β* value is, the larger the values of the standard variations of actual and equalized accessibility are. The reason is that larger *β* values imply people are only willing to accept a shorter travel distance to obtain service from RCFs. Larger *β* values, however, lead to smaller ratios between actual and equalized accessibility standard variation. The ratio reaches a high level of 3.72 when *β* is 0.6 and it decreases to 1.73 when *β* is 1.4. In the following sections, we will discuss the optimized result of the scenario setting *β* as 1.0, which was also adopted in our previous study [12].A relatively large number of beds (51% of the total) are optimally located in the suburban districts between the central and peripheral districts of Beijing (Figures 2, 3). A smaller number of beds (33% and 16% of the total) are optimally located in the central and peripheral districts respectively. By contrast, the distribution of the elderly population is different from the optimized distribution of residential care resources as shown in Figures 2 and 3. The percentage of the elderly population by district declines from the central districts outwards.

**Table 2:** Actual and equalized standard variation of accessibility to RCFs in Beijing

***β***	**Actual standard variation of accessibility**	**Equalized standard variation of accessibility**	**Ratio**
0.6	0.0153	0.0041	3.7163
0.8	0.0175	0.0051	3.4666
1.0	0.0207	0.0066	3.1275
1.2	0.0243	0.0084	2.8982
1.4	0.0280	0.0162	1.7312

The optimized results imply that more residential care resources (34,762 beds, 51% of the total) should be located in the suburban districts to maximize equitable accessibility for the elderly population in Beijing. The results confirm the municipal special plan for the residential care system. There are approximately 1.1 million older people (64% of the total) in the central districts, while the optimized number of beds is only 22,413 (33% of the total). By contrast, the elderly population and optimized number of beds are 0.4 million (25% of the total) and 34,762 (51% of the total) respectively in the suburban districts. The high proportion of demand in the central districts can be met by the residential care resources located in the suburban districts as the travel conditions are relatively convenient in the central districts. Meanwhile, the land resources are relatively more abundant in the suburban districts than in the central districts. RCFs in the peripheral districts mainly meet the demand of the local elderly population, where the elderly population is 0.2 million (11% of the total) and the optimized number of beds is 10,867 (16% of the total).

In the optimized scenario, two subdistricts provide more than 1,000 RCF beds. They are Changxingdian in Fengtai District and Dongxiaokou in Changping District with 1,125 and 1,013 beds respectively. There are 21 subdistricts providing more than 600 RCF beds, among which 6 are in Tongzhou District, 5 in Haidian District, 3 in Daxing District, 2 in Shunyi and Chaoyang Districts, and 1 in Fengtai, Fangshan and Changping Districts respectively. These subdistricts should be the main areas for the allocation of RCFs beds. By contrast, 29 subdistricts provides less than 10 RCF beds, among which 10 RCFs are in Dongcheng and Xicheng Districts, 5 are in Chaoyang District, 2 in Haidian District, and 1 in Haidian and Fengtai Districts. In reality, it would not make economic sense to allocate any beds in these subdistricts because of the small number of optimized RCF beds and the cost of building facilities for such small numbers of beds.

**Figure 4:**
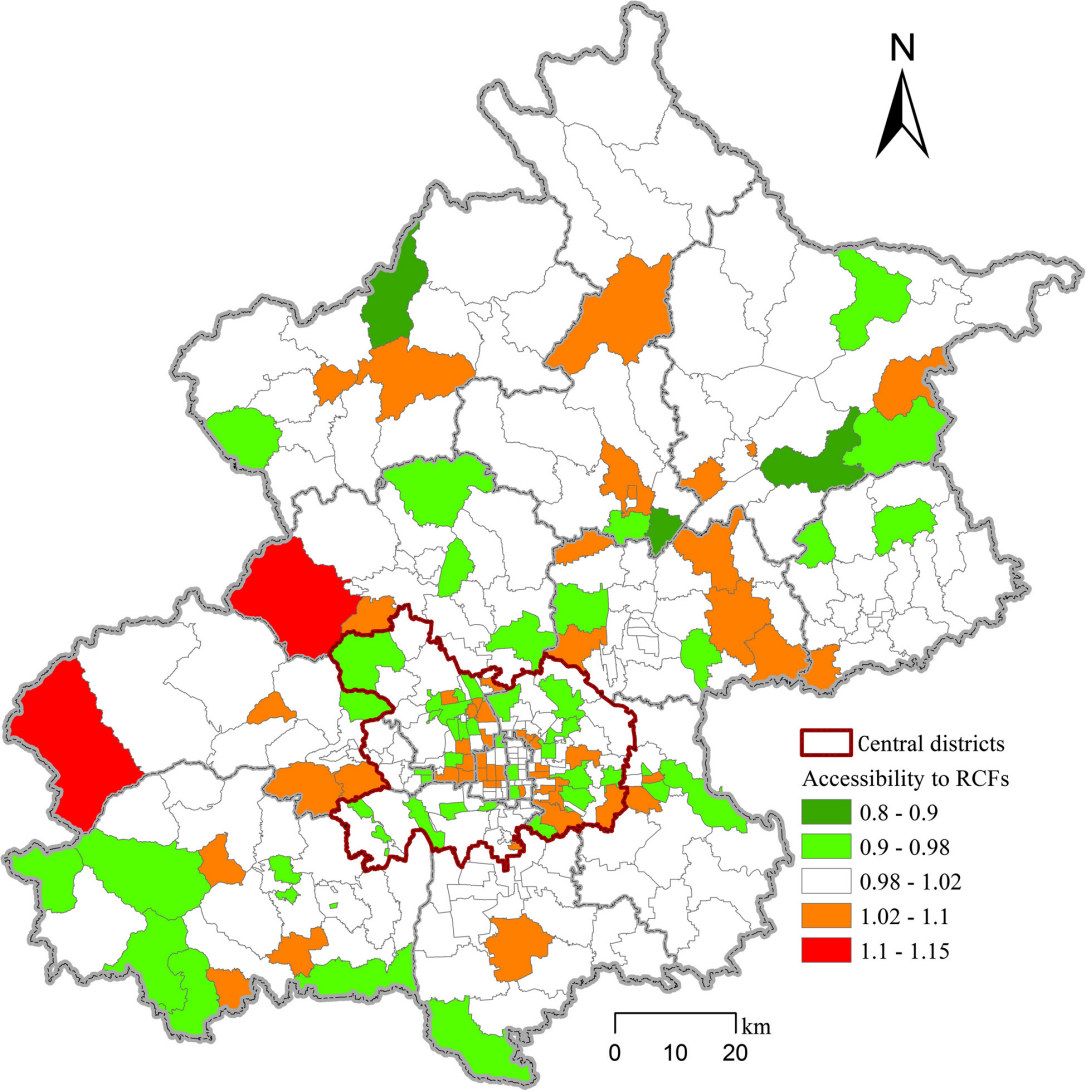
Spatial distribution of the optimized accessibility equity to RCFs.

The optimized RCF bed numbers at the district level are shown in Table 3. Daxing, Tongzhou, Chaoyang, Haidian, Changping, Fangshan and Shunyi Districts have more than 5,000 RCF beds each and they are all located in suburban districts. These regions do not only need to meet the local demands for residential care services but also the demands from the central districts. Dongcheng and Xicheng Districts, which are in the central district, have only 489 RCF beds in total, in stark contrast to the large number of elderly people (270,786 in total) living in these areas. The peripheral districts of Beijing, including Yanqing, Pinggu, Miyun, Huairou and Mentougou Districts, however, mainly provide residential care services for the local elderly population.The optimized results greatly improve equal access to residential care resources. The optimized layouts have a very low accessibility standard variation (0.0066), which means that the inequality in accessibility of demand locations to RCFs is insignificant, while the actual accessibility standard variation (0.0207) is 3.1275 times that of the optimized results. As the goal of the optimization is to minimize the accessibility differences at the subdistrict level, we evaluated the results by using the optimized accessibility score divided by 0.04 (the weighted average accessibility, see formula (4)). Figure 4 shows the results of the evaluation of the comparison between the optimized accessibility scores and the weighted average accessibility at the subdistrict level. The results show that the score in most subdistricts is between 0.98 and 1.02, which is very close to the weighted average accessibility measure. The subdistricts with scores that are higher or lower than the weighted average accessibility score are relatively evenly distributed both in the central and peripheral districts, which imply that the effects of our optimized results are positive and reliable.

**Table 3:** The optimized RCF bed numbers at the district level in Beijing

**District**	**RCF bed number**	**District**	**RCF bed number**
Daxing	8197	Mentougou	3851
Tongzhou	7779	Shijingshan	2280
Chaoyang	7578	Huairou	2117
Haidian	7510	Miyun	1924
Changping	6803	Pinggu	1864
Fangshan	6762	Yanqing	1505
Shunyi	5221	Dongcheng	143
Fengtai	4555	Xicheng	346
Total		68437	

### The gaps between the actual and optimal layouts

**Figure 5:**
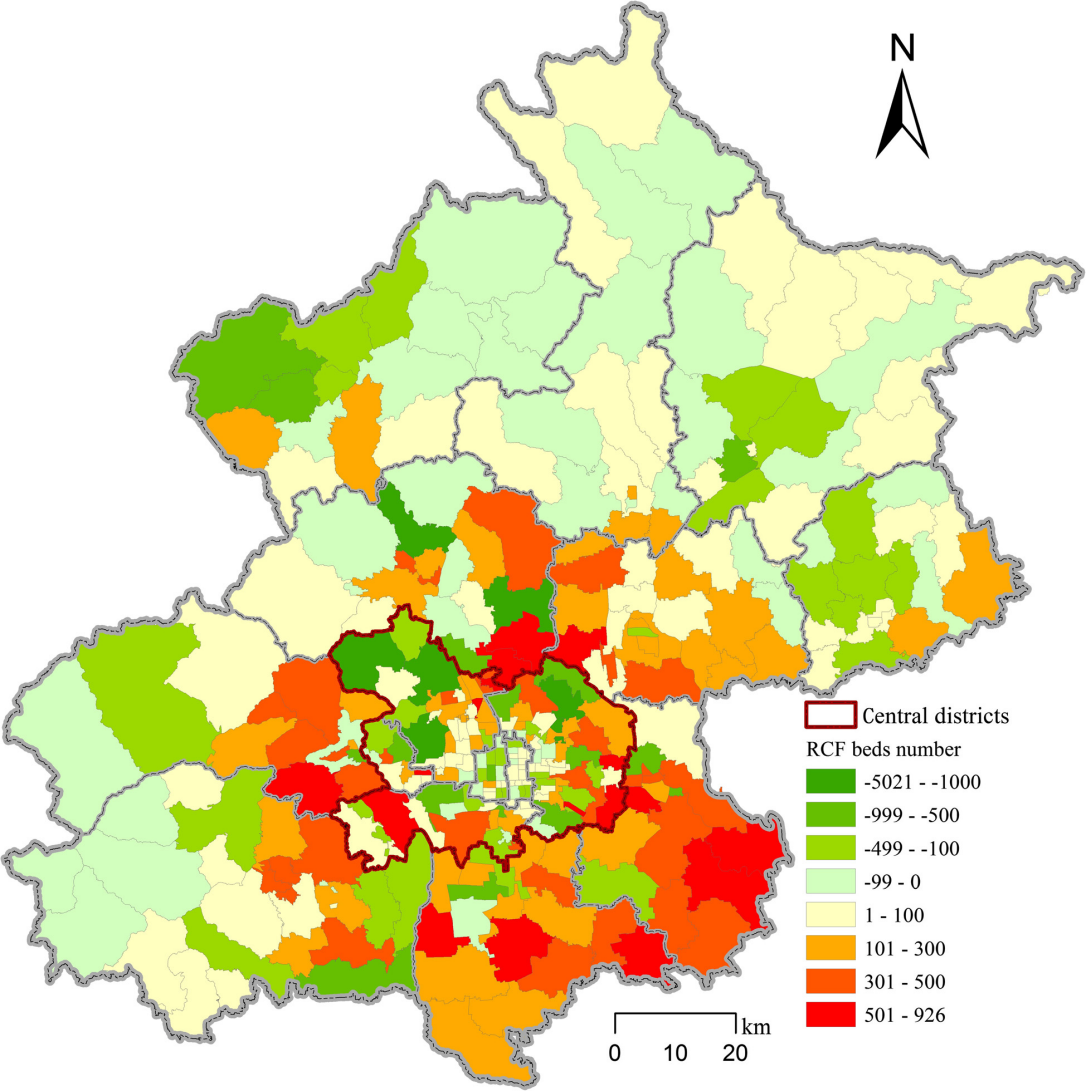
The gaps between optimal and actual number of RCF beds at the subdistrict level.

As we mentioned at the beginning, the goal of the official special plan on the residential care system is set to provide residential care resources for four percent of the total elderly population. We estimated the demands based on this goal and compared the existing residential care resources with the optimized results at the subdistrict level. We calculated the differences between the optimal number and the actual number of RCF beds in each subdistrict (Figure 5). If the value is positive, more beds should be allocated in the subdistrict; otherwise, there are redundant beds that should be reallocated to other districts to achieve an optimal distribution of RCFs. The results provide suggestions for future allocation of RCF beds at the subdistrict level.

According to the policy goal that 4 percent of the elderly population will live in RCFs, 68,437 RCF beds are needed to meet the demands based on the elderly population in 2010. The existing number of RCF beds was 69,314 in 2013, which already meets the total demand for RCF beds in 2010. The optimization results show that more beds are needed in the suburban districts (5,961 beds). There are 30 subdistricts that need more than 400 new RCF beds: 6 in Tongzhou and Chaoyang, 5 in Daxing, 4 in Mentougou, 3 in Changping, and 1 in Fangshan, Fengtai, Shijingshan and Shunyi Districts respectively.

In some subdistricts located in the central and peripheral districts, the existing RCF beds, however, are redundant compared to the optimization results (5,253 and 1,584 beds respectively). The number of surplus beds is more than 400 in 26 subdistricts, which are located in 13 out of 16 districts (except Dongcheng, Huairou and Shunyi): 5 in Chaoyang, 3 in Changping and Haidian, and 1 or 2 subdistricts in the other districts. That is to say, the oversupply of RCF beds is dispersed in various districts rather than concentrated in a few districts. One of the important reasons for the oversupply of RCF beds in some subdistricts is that many of the beds are concentrated in relatively large RCFs (i.e., facilities with more than 500 beds) near to green spaces in the subdistricts.

## Discussions and conclusions

This paper first measures the accessibility to RCFs at the subdistrict level in Beijing. The spatial optimization model is developed to maximize equal access to residential care resources by minimizing the total square difference between the accessibility score of each demand location and the weighted average accessibility measure. The standard variation of accessibility at each demand location is 0.0066 in our results. The optimal results show that more RCF beds (34,762 beds, 51% of the total) should be allocated to the suburban districts, and less in the central and periphery districts (22,413 and 11,262 beds, 33% and 16% of the total, respectively).

The results also show the gaps between the existing and optimal layouts, which suggest that RCF beds should be increased in the suburban districts (5,961 beds). The RCF beds in some subdistricts located in the central and peripheral districts, however, are oversupplied (5,253 and 1,584 beds respectively). The gaps imply that the existing large supply in some subdistricts seems irrational under the maximum equity objective. The oversupply in most subdistricts with large RCFs is considerable compared to the existing bed numbers. For example, the Shisanling Township in Changping District had 5,350 RCF beds in 2013. It was the subdistrict with the largest absolute number of beds among all the subdistricts, but the optimal number is only 329. The RCFs in 24 of the 30 subdistricts that have more than 600 RCF beds are oversupplied for more than half of the existing RCFs beds. More efforts, however, are needed to confirm whether the existing RCF clusters are irrational indeed. On-site investigations to find out if the occupancy rate of RCFs located in existing clusters is low would be helpful for confirming the over-supply suggested by the modeling results.

The Beijing Municipal Commission of Urban Planning and the Beijing Civil Affairs Bureau issued a special plan for the development of RCFs [9]. It claimed that the new increases in RCFs should mainly be allocated in the suburban districts. According to the special plan, 39.8%, 40.8% and 19.4% of RCF beds will be located in central city, new towns and rural areas respectively. Considering that the new towns in the special plan refer to the urban areas of the suburban and periphery districts or counties, planned distribution of RCF beds is quite similar to the results of this study. The plan recommended enlarging existing RCFs or renovating other types of public facilities to make them into RCFs to maximize the potential capacity instead of building new ones in the central districts. New RCFs are planned in the outer region of the central districts to meet local demand. Relatively more new RCFs are planned for the suburban districts with the goal of providing residential care resources for the elderly population living in the central districts. The optimized results of this study correspond to the municipal special plan. The results of this study therefore confirm that the layout proposed will meet the equality in accessibility to RCFs criterion to a great extent.

The optimization objective in this study differs from the traditional optimization studies of facility locations. The former aims to maximize equality in accessibility to facilities by minimizing the disparity in accessibility of each demand location [26], while most of the existing studies aim to maximize the efficiency of the facility allocation. The formulations of the two types of optimization objectives are different. The traditional objectives usually take a linear form, but the maximum equality objective is nonlinear [26]. Both types of optimization objectives are important and significant for the planning of facility locations.

The methods used in this paper also improve on the measurement of accessibility to facilities in optimization studies of facility locations. The traditional location-allocation models usually adopt a dichotomous distance-decay form, namely, accessibility to facilities is assumed to be homogeneous within a certain threshold distance and inaccessible beyond the threshold distance. Moreover, traditional models do not take the capacity of facilities and difference in demand at locations into account. To measure accessibility more accurately, this study takes a 2SFCA form with a distance-decay function.

This research is one of the first studies to apply the optimization model maximizing equity in accessibility to residential care facilities considering different utilization behaviors in residential care services and health care services. The PSO method is used rather than a traditional quadratic programming method to solve the location optimization problem, which is found to be more effective than the traditional method. We also compare the optimization results with the distribution of RCF beds proposed by the special plan. The results show that the government’s plan on RCFs will meet the equality in accessibility to RCFs criterion to a great extent.

There are some limitations to this study. First, the geographical and socio-economic factors of elderly people’s preferences for RCFs are not considered in the optimization model. Second, the most recent population census data are only available for 2010, and the smallest scale of census data is only available at the subdistrict level (the average number of elderly population in subdistricts is 5,268). The census data at a smaller geographical scale would improve the accuracy of our findings.

In the future, we will strive to obtain smaller-scale or predicted elderly population data for better optimization results. Moreover, various optimization objectives (both maximum equality and efficiency) will be adopted to compare optimization results and costs in various optimization scenarios. As a first step, the findings of the current research, however, offer support for knowledge-based policy-making and planning of residential care facilities in Beijing, as well as in other regions in China and developing countries, where there is still in a great shortage of facilities and services for the elderly population.

## Abbreviations

2SFCA: Two-step floating catchment area; OLS: Ordinary least square; PSO: Particle swarm optimization; QP: Quadratic programming; RCF: Residential care facility.

## Competing interests

The authors declare that they have no competing interests.

## Authors’ contributions

ZT and YC contributed to the design and analysis of the study and writing of the manuscript. TD contributed on the design and analysis of the study and revising the manuscript. MWR contributed to drafting the manuscript and revising it critically. All authors read and approved the final manuscript.
